# Transcranial ultrasound as a diagnostic tool for hypoxic-ischemic encephalopathy in preterm neonates: A cross-sectional study

**DOI:** 10.6026/973206300214785

**Published:** 2025-12-15

**Authors:** Riya Agarwal, Nisha Rai, Rajeev Kumar Ranjan, Suresh Kumar Toppo, Anima Ranjni Xalxo, Manisha Oraon, Harish S Gupta, Md Shahrukh Ansari

**Affiliations:** 1 Department of Radio-diagnosis, Rajendra Institute of Medical Sciences, Ranchi, Jharkhand, India

**Keywords:** Hypoxic-ischemic encephalopathy, preterm neonates, transcranial ultrasound, diagnostic accuracy, neurosonography

## Abstract

Hypoxic-ischemic encephalopathy (HIE) is a major cause of morbidity and mortality in preterm neonates, necessitating early and accurate diagnosis. Therefore, it is of interest to evaluated the diagnostic accuracy of transcranial ultrasound (TUS) in 168 preterm neonates with clinical features of HIE. Significant ultrasound findings-such as intraventricular hemorrhage, ventriculomegaly, multicystic leukomalacia and abnormal Doppler indices-showed strong correlations with severe SARNAT stages. The combined parameters demonstrated high diagnostic accuracy, with a sensitivity of 85.7% and specificity of 82.4%. TUS proves to be an effective, accessible tool for early detection and prognostication of HIE in preterm neonates.

## Background:

Hypoxic-ischemic encephalopathy (HIE) is a serious condition resulting from decreased oxygen and blood flow to the brain around the time of birth, leading to significant neurological morbidity and mortality in neonates [1]. The incidence of HIE in preterm neonates ranges from 5-9 per 1000 live births, which is substantially higher than the 1.5 per 1000 live births observed in term infants [2]. This difference is attributed to the vulnerability of the developing brain in preterm infants, particularly the periventricular white matter, which is susceptible to hypoxic-ischemic injury due to its vascular supply and high metabolic demands [3]. The diagnosis of HIE in preterm neonates presents unique challenges compared to term infants. While standard neurological examination is applicable in term neonates, diagnosing HIE in preterm neonates is complicated by physiological immaturity, which can obscure clinical features [4]. Additionally, the patterns of brain injury differ between preterm and term infants, with preterm neonates more commonly exhibiting periventricular leukomalacia and germinal matrix hemorrhages, while term infants typically show parasagittal watershed infarcts and deep gray matter injury [5]. Magnetic resonance imaging (MRI) is considered the gold standard for evaluating neonates with suspected brain injury [6]. However, its limited availability, high cost and requirement for sedation make it impractical for routine screening of all at-risk neonates. In contrast, transcranial ultrasound (TUS) is widely available, portable, cost-effective and can be performed at the bedside without sedation [7]. These advantages make TUS an attractive option for initial screening and monitoring of preterm neonates at risk for HIE. Transcranial ultrasound utilizes the acoustic windows provided by the open fontanelles in neonates to visualize brain structures and pathology [8]. The anterior fontanelle is the primary window, allowing for both coronal and sagittal views, while the mastoid and posterior fontanelles provide additional access to evaluate the brainstem, posterior subcortical white matter and cerebellum [9]. TUS can detect various abnormalities associated with HIE, including increased parenchymal echogenicity, ventriculomegaly, intraventricular hemorrhage and cystic encephalomalacia [10, 11- 12]. Despite these advances, there remains a research gap regarding the diagnostic accuracy of TUS specifically for HIE in preterm neonates and the correlation between ultrasound findings and clinical severity. While several studies have examined TUS findings in preterm infants, few have focused specifically on HIE or established clear correlations between ultrasound parameters and clinical staging using standardized systems like the SARNAT classification. Therefore, it is of interest to establish the importance of transcranial ultrasonography as a diagnostic modality in preterm neonates with clinical features of HIE, to document the morphological changes associated with HIE in brain parenchyma and to determine the association between these morphological changes and clinical features.

## Materials and Methods:

## Study design and setting:

A hospital-based cross-sectional study was conducted over a period of 12 months (December 2023 to December 2024) in the Department of Radiodiagnosis, Rajendra Institute of Medical Sciences (RIMS), Ranchi, Jharkhand. The study included preterm neonates admitted to the Department of Pediatrics with suspected or confirmed HIE.

## Sample size:

Based on the records maintained in the Department of Pediatrics, RIMS, Ranchi, approximately 14 preterm neonates suspected of HIE are admitted per month. Considering a 12-month study period, the sample size was calculated to be 168 cases.

## Eligibility Criteria:

## Inclusion criteria:

[1] All preterm neonates born in RIMS, Ranchi (before 37 completed weeks of gestation) suspected or clinically confirmed of HIE:

1) Persistence of APGAR Score 0-3 for >5 minutes

2) Requirement of positive pressure ventilation

3) Profound metabolic or mixed academia (pH < 7) in umbilical artery blood sample if obtained

4) Mild, moderate, or severe HIE, as defined by Sarnat and Sarnat Staging

[2] Preterm neonates born in RIMS, Ranchi for whom complete medical records, including relevant clinical information and follow-up data, were available for analysis.

## Exclusion criteria:

[1] Neonates whose parents or legal guardians did not provide informed consent for participation in the study

[2] Neonates born after a gestational age of 37 weeks

[3] Major congenital malformation, chromosomal abnormalities, or any other metabolic disorders

[4] Preterm neonates with known pre-existing brain pathologies or conditions that may interfere with the interpretation of transcranial ultrasound findings or the assessment of HIE

[5] Cases lost to clinical follow-up

[6] Preterm neonates suspected of HIE but born outside RIMS, Ranchi

## Ethical considerations:

Ethical clearance was obtained from the Institutional Ethics Committee of RIMS, Ranchi. The study adhered to the tenets of the Declaration of Helsinki. Written informed consent was obtained from the parents or legal guardians of all preterm neonates included in the study.

## Clinical assessment:

Detailed clinical history including gestational age, birth weight, Apgar scores, clinical signs and other pertinent information was recorded for each neonate. Clinical staging of HIE was performed using the SARNAT classification system, which categorizes HIE into three stages (mild, moderate, severe) based on the level of consciousness, spontaneous activity, posture, muscle tone, primitive reflexes and autonomic reflexes.

## Transcranial ultrasound procedure:

Transcranial ultrasound was performed using a Philips Affini 70G machines equipped with high-frequency transducers suitable for neonatal imaging. A small footprint curvilinear probe (5-8MHz) and a linear high-frequency probe (4-18MHz) were used for the examinations.

The following features on transcranial ultrasonography were evaluated for the diagnosis of HIE:

[1] Increased cerebral parenchymal echogenicity

[2] Slit-like ventricles

[3] Diffuse parenchymal echoes

[4] Multi-cystic leukomalacia

[5] Intracranial hemorrhage

[6] Ventriculomegaly

[7] Echogenic deep gray nuclei and cerebellum

[8] Abnormal doppler indices

All transcranial ultrasound scans were performed with the neonate lying supine with the head held in a neutral position. The anterior fontanelle was primarily used as the acoustic window, through which a minimum of 5 sagittal and 6 coronal sweeps were obtained. Gray-scale images were acquired and doppler indices of the anterior cerebral arteries were obtained on sagittal images. Additional images were obtained through the mastoid fontanelle by placing the probe 1 cm above and behind the tragus for the acquisition of axial images. Safety measures undertaken to minimize potential risk to the neonates included using an adequate amount of coupling gel, maintaining gentle pressure on the fontanelles and closely monitoring the neonate's vital signs throughout the procedure.

## Statistical analysis:

The data were analyzed using appropriate statistical methods. Fischer Exact Test was used to determine the significance of the study, classifying the parameters in a 2x2 table. Diagnostic statistics including sensitivity, specificity, positive predictive value (PPV), negative predictive value (NPV) and p-value were computed to find the correlation with clinical outcome. A p-value of less than 0.05 was considered statistically significant. Statistical analysis was performed using SPSS version 25.0 (IBM Corp., Armonk, NY, USA). intraventricular hemorrhage, 15 had Grade I, 8 had Grade II, 6 had Grade III and 3 had Grade IV hemorrhage. Among the 25 neonates with multi-cystic leukomalacia, 12 had Grade I, 7 had Grade II, 4 had Grade III and 2 had Grade IV. The correlation between transcranial ultrasound findings and clinical features (SARNAT staging) is presented in Table 3 (see PDF).

## Results:

A total of 168 preterm neonates with suspected or confirmed HIE were included in the study. The demographic and clinical characteristics of the study population are presented in Table 1 (see PDF). Among the 168 preterm neonates, 95 (56.5%) were male and 73 (43.5%) were female. The majority of neonates (105, 62.5%) were early preterm (<34 weeks gestation), while 63 (37.5%) were late preterm (34-37 weeks gestation). Regarding birth weight, 25 (15%) neonates had normal birth weight (>2500g), 109 (65%) had low birth weight (1500-2500g) and 34 (20%) had very low birth weight (<1500g). Most neonates (131, 78%) were delivered vaginally, while 37 (22%) were born via caesarean section. Maternal-fetal risk factors were present in 74 (44%) neonates and absent in 94 (56%). The APGAR scores at 5 minutes were ≤1 in 25 (15%) neonates, 2 in 57 (34%) and 3 in 86 (51%). According to SARNAT staging, 45 (27%) neonates had stage I HIE, 69 (41%) had stage II and 54 (32%) had stage III. The distribution of transcranial ultrasound findings in the study population is presented in Table 2 (see PDF). Increased parenchymal echogenicity was the most common finding, observed in 138 (82%) neonates, followed by diffuse parenchymal echoes in 99 (59%), slit-like ventricles in 74 (44%) and abnormal doppler indices in 74 (44%). Ventriculomegaly was present in 50 (30%) neonates, intraventricular hemorrhage in 32 (19%), multi-cystic leukomalacia in 25 (15%) and echogenic deep gray nuclei/cerebellum in 15 (9%). Among the 32 neonates with intraventricular hemorrhage, 15 had Grade I, 8 had Grade II, 6 had Grade III and 3 had Grade IV hemorrhage. Among the 25 neonates with multi-cystic leukomalacia, 12 had Grade I, 7 had Grade II, 4 had Grade III and 2 had Grade IV. The correlation between transcranial ultrasound findings and clinical features (SARNAT staging) is presented in Table 3 (see PDF).

## Statistically significant correlations with severe clinical features (SARNAT stage III) were found for:

[1] Intraventricular hemorrhage (p=0.0001): Sensitivity 31%, Specificity 93.8%, PPV 84.3%, NPV 55.8%

[2] Ventriculomegaly (p=0.0007): Sensitivity 42.5%, Specificity 81.8%, PPV 68%, NPV 61%

[3] Multi-cystic leukomalacia (p=0.00): Sensitivity 27.3%, Specificity 95.6%, PPV 84%, NPV 60.8%

[4] Abnormal doppler indices (p=0.00001): Sensitivity 61.2%, Specificity 83%, PPV 85%, NPV 57.4%

No statistically significant correlations were found for increased parenchymal echogenicity (p=0.32), slit-like ventricles (p=0.87), diffuse parenchymal echoes (p=0.15), or echogenic deep gray nuclei/cerebellum (p=0.28).

A combined analysis of the statistically significant parameters (intraventricular hemorrhage, ventriculomegaly, multi-cystic leukomalacia and abnormal doppler indices) showed:

[1] Sensitivity: 85.7%

[2] Specificity: 82.4%

[3] Positive predictive value: 75.8%

[4] Negative predictive value: 83.6%

[5] p-value: 0.00001 (highly significant)

[6] Relative risk: 4.6

## Discussion:

This study aimed to assess the diagnostic accuracy of transcranial ultrasound (TUS) in preterm neonates with suspected hypoxic-ischemic encephalopathy (HIE) and to correlate ultrasound findings with clinical manifestations. The results demonstrate that TUS is a valuable diagnostic tool for HIE in preterm neonates, with specific parameters showing strong correlation with clinical severity. The most common ultrasound finding in our study was increased parenchymal echogenicity (82%), which is consistent with previous reports. Eken *et al.* reported that 26.4% of full-term neonates with HIE had areas of increased echogenicity in the periventricular and/or cortical white matter [13]. The higher incidence in our study may be attributed to the focus on preterm neonates, who are more susceptible to white matter injury due to the vascular anatomy of the developing brain [14]. Intraventricular hemorrhage (IVH) was observed in 19% of our study population, with statistically significant correlation with severe clinical features (p=0.0001). This finding is consistent with previous studies that have reported IVH as a common complication in preterm neonates with HIE. Soni *et al.* found that cranial ultrasound is both sensitive and specific for identifying various types of intracranial hemorrhage in high-risk neonates [15]. The grading of IVH in our study followed the Papile classification, with Grade I being the most common, which aligns with the natural history of IVH in preterm infants [16]. Ventriculomegaly was present in 30% of our study population and showed a significant correlation with severe clinical features (p=0.0007). This finding is supported by Ilves *et al.* who demonstrated that an increase in ventricular size between 21-159 days of age was significantly linked to the severity of HIE and psychomotor development at 18 months [17]. Ventriculomegaly in HIE is generally passive, resulting from progressive necrosis and loss of periventricular white matter, but can also be caused by obstruction due to IVH [18]. Multi-cystic leukomalacia was observed in 15% of our study population and showed a strong correlation with severe clinical features (p=0.00). This finding is consistent with the study by de Vries *et al.* who reported that cystic periventricular leukomalacia is associated with poor neurodevelopmental outcomes in preterm infants [19]. The grading of multi-cystic leukomalacia in our study followed the classification by Kuban *et al.* with Grade I being the most common [20]. Abnormal doppler indices of the anterior cerebral artery were observed in 44% of our study population and showed the strongest correlation with severe clinical features (p=0.00001). This finding is supported by several previous studies. Archer *et al.* demonstrated that an abnormal resistance index (RI) of 0.5 or less within the first 72 hours post-birth is highly predictive of a poor prognosis in neonates with HIE [21]. Similarly, Ilves *et al.* reported that both cerebral blood flow velocity and RI values are significantly correlated with the severity of HIE and subsequent neurodevelopmental outcomes [22]. The combination of significant parameters (IVH, ventriculomegaly, multi-cystic leukomalacia and abnormal doppler indices) showed high sensitivity (85.7%), specificity (82.4%), positive predictive value (75.8%) and negative predictive value (83.6%), with a relative risk of 4.6. This suggests that the presence of at least one of these parameters significantly increases the risk of severe clinical manifestations, while their absence can reliably predict milder clinical features. Interestingly, some commonly reported ultrasound findings such as increased parenchymal echogenicity, slit-like ventricles, diffuse parenchymal echoes and echogenic deep gray nuclei/cerebellum did not show statistically significant correlations with clinical severity in our study. This may be due to the high prevalence of these findings in both mild and severe cases, reducing their specificity for predicting clinical outcomes. Additionally, echogenic deep gray nuclei/cerebellum was observed in only 9% of our study population, which may have limited the statistical power to detect a significant correlation. Our findings have important clinical implications. The strong correlation between specific TUS parameters and clinical severity suggests that TUS can be used as a reliable tool for early diagnosis and prognostication of HIE in preterm neonates. This is particularly valuable in resource-limited settings where advanced imaging modalities like MRI may not be readily available. The portability and bedside applicability of TUS make it an ideal tool for serial monitoring of brain injury in critically ill preterm neonates. The strengths of our study include the relatively large sample size, the standardized protocol for TUS examination and the use of established classification systems for both clinical staging (SARNAT) and ultrasound findings. However, our study has some limitations. First, TUS is operator-dependent and the quality of the examination is influenced by the skill and experience of the sonologist. Second, TUS has lower sensitivity for detecting cortical lesions compared to MRI. Third, our study did not include long-term neurodevelopmental follow-up, which would have provided additional validation of the prognostic value of TUS findings. Future research should focus on longitudinal studies to assess the long-term neurodevelopmental outcomes of preterm neonates with HIE and their correlation with early TUS findings. Additionally, comparative studies between TUS and advanced imaging modalities like MRI would further establish the diagnostic accuracy of TUS in this population. In conclusion, our study demonstrates that transcranial ultrasound is a valuable diagnostic tool for HIE in preterm neonates, with specific parameters including intraventricular hemorrhage, ventriculomegaly, multi-cystic leukomalacia and abnormal doppler indices showing strong correlation with clinical severity. The combination of these parameters can reliably predict clinical outcomes, making TUS an essential modality for early diagnosis and management of HIE in preterm neonates.

## Conclusion:

Transcranial ultrasound proves to be a highly effective, accessible and non-invasive diagnostic tool for identifying hypoxic-ischemic encephalopathy in preterm neonates. Significant ultrasound markers-including intraventricular hemorrhage, ventriculomegaly, multicystic leukomalacia and abnormal Doppler indices-correlate strongly with clinical severity and demonstrate high diagnostic accuracy. Thus, we show TUS as a preferred initial screening modality in both routine and resource-limited neonatal care settings.
